# The Farnesoid X Receptor as a Master Regulator of Hepatotoxicity

**DOI:** 10.3390/ijms232213967

**Published:** 2022-11-12

**Authors:** Magdalena Rausch, Sophia L. Samodelov, Michele Visentin, Gerd A. Kullak-Ublick

**Affiliations:** Department of Clinical Pharmacology and Toxicology, University Hospital Zurich, University of Zurich, 8006 Zurich, Switzerland

**Keywords:** drug-induced liver injury, farnesoid X receptor (FXR), hepatotoxicity, inflammation, liver, NAFLD and NASH

## Abstract

The nuclear receptor farnesoid X receptor (FXR, NR1H4) is a bile acid (BA) sensor that links the enterohepatic circuit that regulates BA metabolism and elimination to systemic lipid homeostasis. Furthermore, FXR represents a real guardian of the hepatic function, preserving, in a multifactorial fashion, the integrity and function of hepatocytes from chronic and acute insults. This review summarizes how FXR modulates the expression of pathway-specific as well as polyspecific transporters and enzymes, thereby acting at the interface of BA, lipid and drug metabolism, and influencing the onset and progression of hepatotoxicity of varying etiopathogeneses. Furthermore, this review article provides an overview of the advances and the clinical development of FXR agonists in the treatment of liver diseases.

## 1. Introduction

The nuclear receptor superfamily includes more than 40 transcriptional regulators that are activated or suppressed in response to ligand availability [1]. The interplay between nuclear receptors through a series of fine-tuned feedback mechanisms is pivotal in maintaining cellular and organ homeostasis as well as in the onset and progression of a number of diseases [2]. Nuclear receptor signaling is considered particularly relevant to liver homeostasis and pathophysiology [3]. Among others, the nuclear receptor farnesoid X receptor (FXR), encoded by the *NR1H4* gene, has been extensively studied with respect to onset, progression of and recovery from liver disease and hepatic injury [4,5,6]. Two alternative promoters, each containing an internal cryptic splice site, regulate the expression of FXR [7]. Thus far, four isoforms called FXRα1, FXRα2, FXRα3 and FXRα4 have been well characterized, each with unique gene transactivation capacity and selectivity [7,8]. In the human liver, predominant expression of the shorter 1 and 2 isoforms has been observed, where isoforms 3 and 4 are found in a higher degree in intestines and the kidney [8,9]. Apart from the core DNA-binding domain, FXR consists of a ligand-independent transcriptional activation domain, a hinge region, a C-terminal ligand-binding domain and a ligand-dependent activation domain [10]. FXRα1 and FXRα3 have four additional amino acids (MYTG—methionine-tyrosine-threonine-glycine) located between the DNA-binding and hinge domains, exhibiting overall less transcriptional activity than isoforms 2 and 4 [11,12]. Recently, four novel splice variants of the human FXR have been identified in hepatocytes (α5 to α8), all exhibiting complete loss of transactivation activity and only minimal DNA-binding activity [13].

FXR activation was originally characterized using the acyclic sesquiterpene alcohol farnesol as a ligand [14]. Shortly thereafter, FXR was found to bind with the highest affinity to bile acids (BAs) [15,16,17]. Other low affinity FXR ligands are lanosterol [18], androsterone [19], forskolin [20] and cafestol [21]. Canonical FXR signaling is initiated upon ligand binding to FXR, nuclear translocation, and heterodimerization with retinoid X receptor (RXR), which leads to binding to FXR response elements (FXREs) of an array of genes encoding proteins involved in the uptake, de novo synthesis and efflux of BAs, to the global effect of reducing intracellular BA levels. The canonical FXR response element motif contains a single inverted repeat of the hexameric sequence AGGTCA (or similar) separated by a single nucleotide spacer (inverted repeat 1), while multiple additional FXREs have been shown to be targeted by FXR and homo- and heterodimers thereof [22,23]. FXR has been shown to bind DNA and regulate gene transcription independently of RXR, by ligand-initiated homodimerization, heterodimerization with other nuclear receptors including liver receptor homolog-1 (LRH-1) or acting potentially as a monomer, once bound to endogenous or synthetic ligands [13,24,25,26,27].

BA metabolism is intimately connected to lipid homeostasis, thus FXR is an important modulator of lipid metabolism as well, and has gained attention as a therapeutic target in the treatment of non-alcoholic fatty liver disease [28,29,30,31]. Because a number of transporters and enzymes involved in BA and lipid metabolism that are regulated by FXR are shared with the detoxification branch of hepatic metabolism, FXR is also cardinal in the hepatic handling of drugs and xenobiotics, and the kinetics and safety profiles thereof.

This review summarizes the multifaceted functions of FXR in orchestrating cellular homeostasis, balancing metabolism and inflammation in response to toxic intracellular concentrations of BAs and lipids. Finally, it outlines the role of FXR in the hepatic accumulation and metabolism of drugs and the toxicologic ramifications thereof.

## 2. The Role of FXR in Bile Acid Homeostasis and Cholestasis

**Figure 1:**
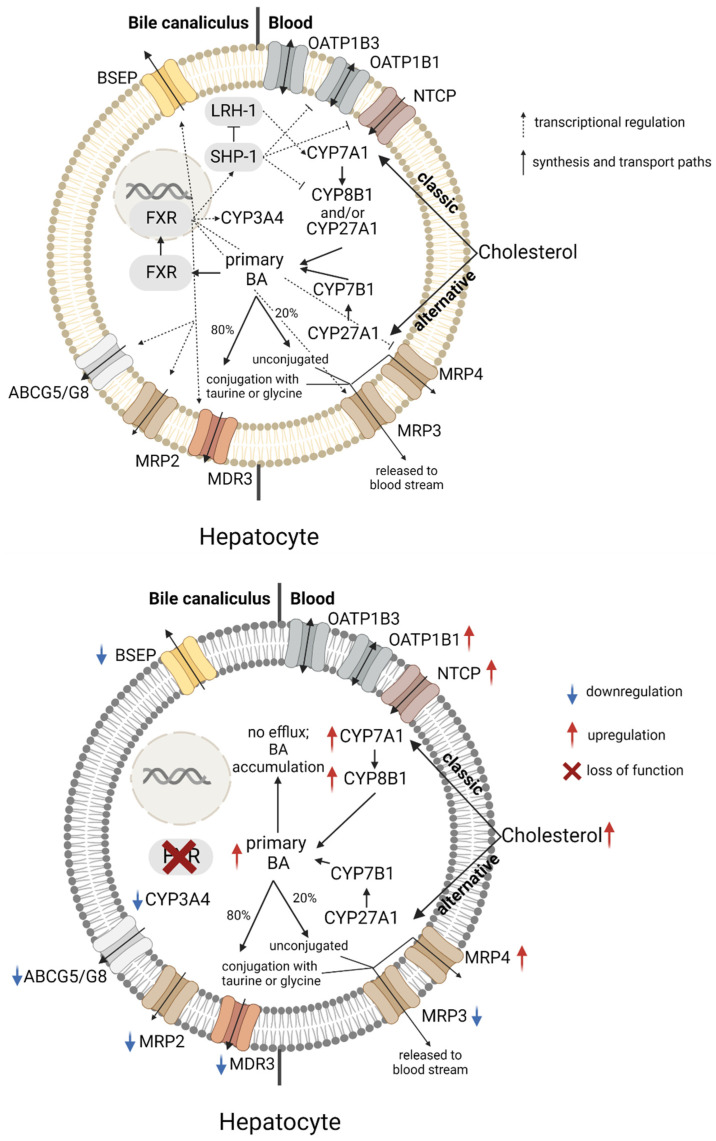
Schematic representation of the regulatory role of FXR in bile acid homeostasis. *Top panel:* Under physiological conditions, hepatocytes convert cholesterol to bile acids following the classic or alternative pathway. Bile acids can bind to and activate FXR, which translocates to the nucleus, where it induces the transcription of genes encoding proteins involved in BA synthesis, transport and degradation, in order to maintain sub-toxic BA intracellular levels. *Bottom panel:* Reduced expression and/or function of FXR leads to an increased BA synthesis, and reduced BA efflux and degradation. ABCG = ATP-binding cassette subfamily G, BA = Bile acid, BSEP = Bile salt export pump, CYP = Cytochrome P450, FXR = Farnesoid X receptor, LRH-1 = Liver receptor homolog 1, MDR = Multidrug resistance protein, MRP = Multidrug resistance associated protein, NTCP = Sodium-taurocholate cotransporting polypeptide, OATP = Organic anion transporter protein, SHP-1 = Small heterodimer protein-1; Figure has been created with BioRender.com.

Bile acids (BAs) are amphipathic, detergent molecules highly concentrated in the bile and responsible for the emulsion and absorption of fats as well as lipophilic vitamins in the small intestine, and for the elimination of excess cholesterol, preventing its precipitation in the bile [32]. In the liver, BAs are synthesized from cholesterol through the classic (neutral) pathway primarily, in which cholesterol is oxidized to 7-α-hydroxycholesterol by the cholesterol 7-α-monooxygenase (CYP7A1, Figure 1), the rate-limiting enzyme in BA de novo synthesis. 7-α-hydroxycholesterol is further oxidized by the CYP8B1 and/or the CYP27A1 to generate the primary BAs cholic acid and chenodeoxycholic acid (CDCA). A minor fraction of cholesterol can enter the alternative (acidic) pathway by direct interaction with CYP27A1 (Figure 1), forming 27-hydroxycholesterol, which is further oxidized by the CYP7B1 to generate CDCA. Primary BAs undergo modification in the hepatocytes by conjugation to taurine or glycine forming conjugated BAs, i.e., bile salts, which are characterized by a lower pk_a_, hence a higher hydrophilicity and a lower cytotoxicity than their unconjugated forms [33]. A minor part of BAs is glucoronidated by uridine diphosphate-glucuronosyltransferases (UGTs), mainly UGT2B4 [34]. The conjugation reactions are highly efficient, however, about 20% of the BA pool skip the conjugation reaction and are present in an unconjugated form in the blood [35,36]. BAs are secreted from hepatocytes into the bile canaliculi by the bile salt efflux pump (BSEP) and accumulate in the gallbladder, from where they are released into the duodenum after a meal [37]. BAs can also flux back into the blood stream through the activity of the multidrug resistance associated proteins (MRP) 3 and 4 [38,39,40]. In the gut, the microbiota deconjugate and hydrolyze BAs to secondary bile salts (e.g., deoxycholic acid) that are reabsorbed through the apical bile salt Na^+^-dependent transporter (ASBT). The vectorial transport of BA from the intestinal lumen to the portal circulation is completed by the OSTα/β transporter, located on the basolateral membrane of hepatocytes [41] and enterocytes [42]. The uptake of BAs from the blood into the hepatocytes is mainly mediated by the Na^+^-taurocholate cotransporting polypeptide (NTCP) and the Na^+^-independent OATP1B1 and OATP1B3. Degradation of BAs is primarily mediated by the cytochrome P450 3A4 (CYP3A4) [43,44,45,46].

A reduction in bile secretion and flow is termed cholestasis (Figure 1). There are two types of cholestasis: hepatocellular and obstructive. Hepatocellular cholestasis originates from a functional impairment of the hepatocytes in the production and/or secretion of the bile components, whereas obstructive cholestasis is defined as a decrease in bile flow due to a physical obstruction of bile flow through intra- or extrahepatic bile ducts. Hepatocellular cholestasis results in the alteration of the physical-chemical equilibrium between cholesterol, phospholipids and BAs, the three main bile components. An increased cholesterol:BA or phospholipid:BA ratio can result in cholesterol crystallization and finally in cholelithiasis. A decreased phospholipid:BA ratio reduces the protective effect of phospholipids against the detergent activity of BAs, which increases the risk of hepatocellular and/or bile duct injury [47]. The concerted action of efflux pumps and flippases expressed on the canalicular membranes of hepatocytes is crucial in bile formation. BSEP and ABCG5/G8 mediate the transport of BA and cholesterol, respectively, MDR3 and *p*-type ATPases translocate phospholipids from the inner to the outer leaflet of the canalicular membrane. Mutations in the *ATP8B1*, *ABCB11* and in the *ABCB4* genes encoding ATP8B1, BSEP and MDR3 respectively, have been linked to progressive familial intrahepatic cholestasis [48]. Loss of function mutations of BSEP are characterized by increased intracellular levels of BAs and a decreased bile flow [49] (Figure 1). Bile duct injuries develop in whole-body MDR3^-/-^ mice with features resembling those observed in human sclerosing cholangitis, suggesting that formation of “toxic” bile could also play an important role in the pathogenesis of various cholangiopathies in humans [47]. Finally, some *ABCG8* genetic variants have been associated with an increased risk of gallstones [50]. Inborn errors of bile acid synthesis have been identified in many of the enzymes involved in bile acid synthesis and extensively reviewed [51]. Among others, deficiency of CYP7A1 (Figure 1) due to a frameshift nonsense mutation, reported in one family, causes increased hepatic cholesterol content and premature gallstone disease. Mutation of the *CYP7B1* gene (Figure 1) in infants causes severe neonatal cholestasis and fibrosis [52].

Upon BA binding to FXR, FXR-RXR heterodimers bind to FXREs in *BSEP* and *CYP3A4* genes, inducing expression of BSEP and CYP3A4 (Figure 1), thereby stimulating BA efflux and degradation, respectively [46,53,54]. FXR, furthermore, induces the expression of the small heterodimer partner 1 (SHP-1) (Figure 1), an atypical member of the nuclear receptor family that lacks a DNA-binding domain but can inhibit the activity of other transcription factors such as the LRH-1 and the hepatocyte nuclear factor 4α (HNF4α), nuclear receptors that induce a the transcription of a variety of genes, including those encoding OATP1B1, CYP7A1, CYP8B1 and NTCP [55,56,57]. The FXR/SHP-1 signaling axis also seems to be dominant in BA-induced repression of NTCP, whereas FXR-mediated repression of OATP1B1 is promiscuous. SHP-1 directly inhibits HNF4α-mediated transactivation of HNF1α, which is central in OATP1B1 transcription. However, HNF4α transcription and nuclear binding activity is decreased by BAs also in a SHP-1-independent manner [46,58]. Intestinal FXR also plays a key role in the downregulation of hepatic CYP7A1 and CYP8B1. The main FXR target gene in the gut is fibroblast growth factor (FGF15 in rodents; FGF19 in humans), which is an enterokine secreted into the portal circulation upon BA stimulation. FGF15/19 reaches the liver, where it activates the FGF receptor 4 (FGFR4). FGFR4 activation is followed by the phosphorylation of the c-Jun N-terminal kinase and thereby the signaling for CYP7A1 and CYP8B1 downregulation [59]. Finally, FXR activation also fine tunes bile formation by transactivation of the transcription of the human *ABCB4* (encoding MDR3) [60] and *ABCG5* genes [61], encoding transporters responsible for the uptake of phosphatidylcholine and cholesterol, respectively. While FXR activation has an overall BA-lowering effect, in vivo studies suggest that the role of FXR in cholestasis very much depends on the etiopathogenesis of the cholestasis. A whole-exome sequencing study detected two mutations (p.R176*, Tyr139_Asn140insLys) in the *NR1H4* gene (encoding FXR) in two unrelated families with neonatal cholestasis. The p.R176* causes premature termination of the protein in the DNA-binding domain, the in-frame insertion p.Tyr139_Asn140insLys resulted in an unstable protein, not detectable at the immunohistological analysis of liver biopsy [62]. Furthermore, four polymorphisms in the *NR1H4* gene have been associated with increased risk for intrahepatic cholestasis of pregnancy [63]. Conversely, studies in whole-body FXR^-/-^ mouse demonstrated that lacking FXR protects against bile duct ligation-induced obstructive cholestasis, possibly because the elevated levels of MRP4, whose basal expression is repressed by FXR, are likely to increase the capacity of BA efflux towards the blood, thereby compensating the impaired canalicular exit [64,65].

## 3. FXR, Fatty Acid Metabolism, and Lipotoxicity

### 3.1. Fatty Acid Synthesis and Metabolism

**Figure 2:**
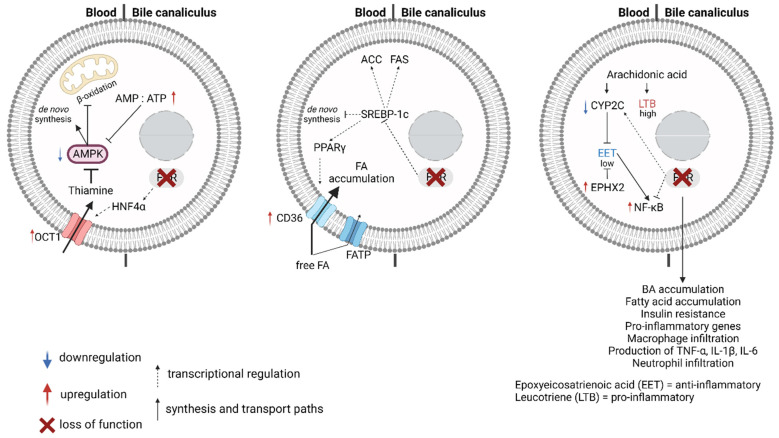
The role of FXR in hepatic lipid accumulation and toxicity. *First panel:* Alterations in thiamine uptake and AMPK activation correlate with NAFLD and liver disease. *Second panel:* Dysregulation of lipid metabolism causing a toxic intrahepatic accumulation of fatty acids leads to NAFLD and correlates to a low expression of FXR. The cellular influx of triglycerides increases and the conversion to fatty acids is enhanced. *Third panel:* In hepatocytes, FXR maintains the equilibrium between epoxyeicosatrienoic acid and leukotriene. ACC = Acetyl-CoA carboxylase, ACOX1 = 2-trans-enoyl-CoAs by the acyl-CoA oxidase 1, AMP = Adenosine monophosphate, AMPK = AMP-activated protein kinase, ATP = Adenosine triphosphate, CD36 = Fatty acid translocase, CPT1 = Carnitine palmitoyltransferase-1, FA = Fatty acid, EPHX2 = Epoxide hydrolase 2, FABP = Fatty acid binding protein, FAS = Fatty acid synthetase, FATP = Fatty acid transport protein, FXR = Farnesoid X receptor, HNF4α = Hepatocyte nuclear factor 4 alpha, NAFLD = Non-alcoholic fatty acid liver disease, OCT1 = organic cation transporter 1, PPARγ = Peroxisome proliferator-activated receptor gamma, SREBP-1c = Sterol regulatory element-binding protein 1c; Figure has been created with BioRender.com.

Next to cholesterol synthesis and degradation to BAs, the liver plays a key role in the metabolism of saturated (e.g., palmitic acid, stearic acid) and unsaturated (e.g., linoleic acid, arachidonic acid) fatty acids (FAs). The primary source of FAs is the triglycerides absorbed from food. The uptake of FAs in the liver is mediated by fatty acid transport proteins (FATPs) and the fatty acid translocase CD36 (Figure 2) [66]. FAs are also the product of de novo lipogenesis from the acetyl-CoA produced during glycolysis (Figure 2). The reaction commences with the production of malonyl-CoA from acetyl-CoA by the acetyl-CoA carboxylase (ACC). The malonyl-CoA is then the substrate of the type I fatty acid synthase complex (FAS), which elongates the acyl chain to varying length, giving rise to various saturated FAs [67].

Dietary and newly synthesized acyl-CoAs can interact with hepatic fatty acid binding proteins (FABPs) to target the organelles designated to FA oxidation. Mitochondria and peroxisomes contribute to FA β-oxidation, whereas the ω-oxidation takes place in the endoplasmic reticulum (Figure 2). The first step of mitochondrial β-oxidation is the transport of acyl-CoA, especially long chain species, into the mitochondrial matrix by carnitine palmitoyltransferase-1 (CPT1) and CPT2, located across the outer and the inner mitochondrial membranes, respectively. CPT1 is considered rate limiting in mitochondrial β-oxidation [68,69]. The desaturation of acyl-CoAs to 2-trans-enoyl-CoAs by the acyl-CoA oxidase 1 is considered the limiting step in peroxisomal β-oxidation [70]. ω-oxidation, where the oxidation occurs at the level of the ω carbon, is mediated by the CYP4A family and is considered a rescue pathway when β-oxidation is defective [71].

Food-derived and the newly synthesized acyl-CoA can serve as the backbone of polar (e.g., phospholipids) and neutral (e.g., triglycerol) lipids. Acyl-CoA and glycerol-3-phosphate are condensed by the glycerol-phosphate acyl transferase to form lysophosphatidic acid, which is further acylated by the acylglycerol-phosphate acyl transferase to form phosphatidic acid. Phosphatidic acid is dephosphorylated by phosphatidic acid phosphatase to generate diacylglycerol. Diacylglycerol can be further acylated by diacylglycerol acyl transferase to form triglycerol, or can be a substrate of the CDP-choline:1,2-diacylglycerol cholinephosphotransferase or the CDP-ethanolamine:1,2-diacylglycerol ethanolaminephosphotransferase to form phosphatidylcholine and phosphatidylethanolamine, respectively [72]. Finally, acyl-CoA is the substrate of the serine palmitoyltransferase, which catalyzes the formation of the sphingoid base from acyl-CoA and serine, and is considered the rate-limiting step in sphingolipid de novo synthesis [73].

### 3.2. FXR and Non-Alcoholic Fatty Liver Disease (NAFLD)

Non-alcoholic fatty liver disease (NAFLD) is defined as the accumulation of fat in the liver (hepatic steatosis) not related to alcohol consumption. Histologically, when hepatic steatosis manifests with signs of inflammation with hepatocyte injury, with or without fibrosis, NAFLD is defined as non-alcoholic steatohepatitis (NASH) [74]. While genetic predisposition has been reported [75], NAFLD is strongly associated with lifestyles predisposing to insulin resistance, altering the lipid metabolism along the adipose tissue-liver axis. In the adipose tissue, insulin promotes triglycerol synthesis and storage, and inhibits lipolysis. In hepatocytes, it activates glycogen storage and de novo lipogenesis, and inhibits gluconeogenesis. When insulin-resistance develops, adipocytes continue to release FAs that are taken up by the liver and are rapidly oxidized or esterified and stored in lipid droplets and in very-low density lipoprotein (VLDL). When the intracellular level of FAs exceeds lipid droplet and VLDL formation capacity, FAs levels build up and become toxic to hepatocytes, cause oxidative stress, and promote inflammation and fibrosis through a variety of mechanisms; events that facilitate the progression to NASH. The expression and activation state of the fuel-sensing enzyme AMP-activated protein kinase (AMPK) is a major molecular determinant of insulin resistance. AMPK activation by a decrease in the AMP:ATP ratio results in the inhibition of FA synthesis and induction of β-oxidation by phosphorylation of acetyl-CoA carboxylase (ACC) [76]. It has been shown that overall AMPK activation is reduced in livers characterized by steatosis and inflammation [77]. Intracellular thiamine (vitamin B1) levels act as a fuel sensor, thereby regulating AMPK signaling pathways [78]. In the liver, thiamine is taken up by the organic cation transporter 1 (OCT1). Oct1^-/-^ mice, characterized by hepatic thiamine deficiency and constitutive activation of AMPK, display reduced triglyceride levels in the liver and are resistant to high fat diet-induced steatosis [79]. Another important molecular determinant in NAFLD onset and progression is the FA transporter CD36. Clinical studies have shown that CD36 expression level is higher in the liver of NAFLD patients (Figure 2). Consistently, the soluble CD36 level is elevated in the plasma of NAFLD patients and positively correlates with the histological grade of hepatic steatosis [80].

While being a direct BA sensor, FXR must fine tune lipid and BA metabolism to preserve the bile content equilibrium, thereby protecting the liver from BA- as well as lipid-induced toxicity. The role of FXR in obesity, insulin resistance and NAFLD is complex, also because the chromosomal deletion of the FXR gene has been shown to cause fundamental dysregulations in other organs, i.e., the brain, changing neuronal signaling that might be involved in appetite and satiety control [28,81]. Whole body and liver-specific FXR ^-/-^ mice develop steatosis and NASH; however, whole-body but not liver-specific FXR^-/-^ mice are less prone to gain weight and to develop insulin resistance than the wild type animals [82,83,84]. Taken together, the data suggests that the loss of hepatic FXR is sufficient to induce NAFLD/NASH. FXR activation indirectly downregulates the expression of OCT1 via SHP-1 repression of the hepatic nuclear transcription factor HNF4α-mediated transactivation of OCT1 transcription, thereby inducing thiamine depletion and AMPK activation, which inhibits lipogenesis and stimulates FA β-oxidation [79,85]. Furthermore, FXR represses the expression of the sterol regulatory element-binding protein 1c (SREBP-1c, Figure 2) in both a SHP-1-dependent and –independent manner [86,87]. Transgenic mice overexpressing nuclear SREBP-1c in adipose tissues spontaneously develop NASH [88]. SREBP-1c is a transcription factor enhancing de novo lipogenesis and triglycerol synthesis by inducing the expression of ACC, FAS and glycerol-phosphate acyl transferase [89]. Additionally, SREBP-1c induces the expression of the nuclear receptor peroxisome proliferator-activated receptor γ (PPARγ) [90], which transactivates the transcription of the gene encoding for the FA transporter CD36 [91]. Notably, mice with PPARγ liver-specific deletion are resistant to steatosis [92,93,94]. Concomitantly, FXR indirectly promotes FA oxidation. A study focusing on the role of FXR in a mouse model of diabetic nephropathy showed that treatment with the FXR ligand obeticholic acid induced the expression of renal CPT1, ACOX and CYP4A14, all three known to be under the regulation of PPARα, and which would result in increased fatty acid oxidation towards lipid catabolism under conditions of pathological cellular lipid accumulation [95]. High fat diet alone did not increase the expression of these genes in the kidney in this study and the mode of regulation of these genes by activated FXR is unclear and likely indirect. While some evidence has been presented that FXR may directly upregulate PPARα in human hepatocytes and in skate fish over non-canonical FXREs identified in the human and skate *PPARα* promoter regions, no regulation could be confirmed in mice nor could a functional FXRE site be identified in this species [96,97,98]. As FXR and PPARα are known to function as nutrient sensors modulating hepatic homeostasis—simply put, active under fed and fasted physiological states, respectively—it should be noted that not only species differences in the function of these nuclear receptors but also the dysregulation and alterations of the crosstalk between the two pathways under pathological states require much more detailed studies to resolve their relative impact in the context of cellular fat accumulation and the clearance thereof via fatty acid oxidation; the understanding of which would be essential to safely and appropriately target either FXR, PPARα or both in the treatment of NAFLD [99,100,101,102,103]. FXR activation also induces the degradation of 1-deoxysphingolipids, atypical sphingolipids formed when acyl-CoA are condensed with alanine instead of serine. 1-deoxysphingolipids, found elevated in the plasma of patients with NAFLD or diabetes [104,105], are toxic metabolites that accumulate in the mitochondria, causing mitochondrial dysfunction and ER stress [106,107]. FXR induces the expression of the CYP4F family members, hydroxylases involved in the metabolism of long chain fatty acids and currently the only known degradation pathway of 1-deoxysphingolipids [105,108]. Noteworthy, the overall anti-lipogenic effect of hepatic FXR has been reported for FXRα2 but not for FXRα1, the other main hepatic isoform [8].

### 3.3. FXR, Arachidonic Acid Breakdown, and Inflammation

FXR indirectly reduces lipid-induced hepatic inflammation by decreasing intracellular FA levels. However, animal studies demonstrated that FXR also exerts a direct anti-inflammatory effect. Low-density lipoprotein receptor^-/-^ (LDLr^-/-^) mice fed a high fat diet develop steatosis but not NASH, whereas the liver of the LDLr^-/-^/FXR^-/-^ mice are characterized by steatosis and inflammation [109]. FXR^-/-^ mice exposed to lipopolysacchride, have higher hepatic expression of pro-inflammatory cytokines. Consistently, FXR activation diminishes lipopolysaccharide-induced inflammation in wild type animals, inhibiting the migration of macrophages and their secretion of pro-inflammatory cytokines [110,111]. A number of in vivo and in vitro studies support the hypothesis that the anti-inflammatory capacity of FXR depends on the ability of repressing NF-kB signaling [95,112,113,114]. In hepatocytes, FXR regulates arachidonic acid metabolism and the balance between anti-inflammatory (epoxyeicosatrienoic acids, EETs) and pro-inflammatory (leukotrienes, LTBs) arachidonate metabolites. Hepatic gene expression of mice with diet-induced NASH revealed a switch in the expression pattern of arachidonate-partitioning genes, notably the downregulation of members of the CYP2C family, which convert arachidonic acid to EETs, and upregulation of the epoxide hydrolase 2 (EPHX2), which inactivates EETs to dihydroxyeicosatrienoic acids. These mice are characterized by a pro-inflammatory LTB_4_/EET ratio [115]. In the same study, in vitro experiments showed that pharmacological activation of FXR can reprogram arachidonate metabolism by inducing the expression CYP2Cs, thereby favoringthe synthesis of EETs over LTBs. Moreover, it was shown that the FXR-induced repression of NF-kB activation might be EET-dependent [115]. In another study, it has been proposed that the acetylation state of FXR protein, which can be regulated by a number of environmental factors including diet, reduces the FXR sumoylation, preventing the inhibition of NF-κB activity [116]. The role of FXR in Kupffer cells seems to be bi-functional (Figure 2), modulating the cellular metabolic and inflammatory responses depending on the environmental stimuli as well as on the concomitant activation of the G-protein-coupled receptor TGR5 sensitive to bile acids, which is highly expressed in Kupffer cells but not in hepatocytes [117]. Apart from organ-resident immune cells, peripheral blood mononuclear cells such as CD4^+^ and CD8^+^ T cells as well as monocytes and B cells express FXR as evidenced by Schote et al. [118]. In these cells, FXR activation appears to be regulated through cytokines, which occurs in relation to liver surveillance and infiltration. Subsequently, cholesterol and BAs modulate B and T cell mediated immune responses. Other cell types involved in the inflammatory response, such as mast cells, dendritic cells, and natural killer T cells, express FXR and might also contribute to the organ inflammatory state [119,120,121]. For instance, the activation of FXR in mast cells has pro-inflammatory characteristics promoting liver damage. Patients with primary sclerosing cholangitis, an autoimmune liver disease characterized by bile duct destruction, have elevated serum histamine, which suggests a higher density of activated mast cells in the injured organ [122]. Dendritic cells in the liver are of anti-inflammatory and tolerogenic nature, but become pro-inflammatory upon onset of hepatic injury [120]. Studies have revealed that dendritic cells respond to treatment with BA derivatives, which reduce the production of tumor necrosis factor-α and lead to the maintenance of the tolerogenic status (Figure 2) [123]. Similar observations were made for natural killer T cells, where FXR activation reduced the expression of osteopontin, interleukin-1β and interferon-γ (Figure 2) [123]. Overall, the role of FXR in inflammation strongly depends on the considered cell type and the given variation of stimuli in the tissue environment, especially in the frame hepatotoxicity.

## 4. FXR and Drug-Induced Hepatotoxicity

Drug-induced liver injury (DILI) is a rare adverse reaction to drugs, natural products or other xenobiotics that occurs either as a predictable or as an unpredictable event with many medications in everyday use. DILI can be of hepatocellular or cholestatic nature, depending on the characteristics of the perpetrator drug and the molecular mechanism of toxicity thereof [124,125].

### 4.1. FXR and Drug-Induced Hepatocellular Injury

In vitro and in vivo data, mostly gathered from studies with acetaminophen (APAP), indicate that FXR protects against drug-induced hepatocellular injury (Table 1). Whole-body FXR^-/-^ mice are more sensitive to APAP than wild type mice [126]. Interestingly, an increased sensitivity to APAP-induced toxicity was found only in the whole-body FXR K.O.^-/-^, not in hepatocyte-specific or macrophage-specific FXR^-/-^ -mice. One possible explanation is that normal FXR expression and activity in one of the two main liver cell types is sufficient to exert the protective effect. Another possibility is that the intestinal FXR substantially contributes to the protection of the liver from APAP toxicity [126]. Treatment with (i) synthetic FXR agonists inducing FXR activation or with (ii) flavonoids, such as schaftoside or saffron (Table 1), increasing FXR expression protect from APAP hepatotoxicity [127,128]. FXR transcriptional activity is regulated by deacetylation mediated by the class III NAD^+^ dependent histone deacetylase sirtuin 1 (SIRT1) [129,130]. It has been shown that the liver of mice treated with triptolide, a diterpenoide epoxide found in the herbal plant *Tripterygium wilfordii* causing DILI, is characterized by downregulation of FXR and SIRT1 (Table 1). Moreover, triptolide-induced DILI is prevented either by FXR and SIRT1 activation [131]. The data suggest that alteration of the FXR/SIRT1 axis might be involved in DILI. In vivo and in vitro experiments showed that FXR activation protects from valproic acid-induced steatosis, reducing oxidative stress and repressing the PPARγ pathway [132]. Combination treatment with the antibiotics amoxicillin and clavulanic acid frequently leads to DILI with characteristics of obstructive cholestasis, because of an immune-mediated damage to the epithelium of interlobular ducts [124]. Interestingly, experiments in collagen-sandwich cultured human hepatocytes showed that clavulanic acid, but not amoxicillin, altered the expression of a number of genes related to BA synthesis and transport, reducing the intracellular BA level. Further gene reporter experiments in U2OS cells and in primary human hepatocytes indicated that clavulanic acid inhibited FXR activation by synthetic and natural agonists [133].

### 4.2. FXR in Drug-Induced Cholestasis

Drug-induced cholestasis comprises 20–40% of all DILI cases, with an associate mortality as high as 10% [134,135]. In most cases, drug-induced cholestasis is of hepatocellular nature and is the result of impaired bile formation due to the direct inhibition by drugs and/or metabolites of BSEP transport activity, resulting in intrahepatic BA accumulation. Strong inhibition of BSEP is considered one of the mechanisms contributing to the severe cases of hepatotoxicity, which led to market withdrawal of drugs such as the antidiabetic troglitazone and the antidepressant nefazodone, or to black-box warnings for hepatotoxicity such as in the case of the anti-pulmonary arterial hypertension medication bosentan [125]. Currently, in vitro prediction of BSEP inhibition by new molecular entities in development is routinary performed to aid decision making on DILI risk. Recent evidence suggests that inhibition of the phospholipid flippase MDR3, also under the regulation of FXR, might contribute to drug-induced cholestasis. Experiments in LLC-PK1 cells co-expressing human MDR3 and BSEP, and displaying vectorial BA and lipid transport in Transwell^®^ system, demonstrated that drugs inducing cholestasis such as the antifungal azoles are potent inhibitors of BSEP as well as MDR3 [136]. Drug-induced cholestasis is largely, but not only, the result of transporter inhibition. In some instances, drug exposure can trigger an immune-mediated destruction of the biliary epithelium, resulting in obstructive cholestasis [124]. FXR plays a key role in the regulation of bile formation, and it is conceivable that a higher FXR expression level and activation state might contribute to the ability of the cells to maintain sub-toxic intracellular BA levels, either by increasing BSEP and MDR3 capacity, and/or by inducing the MRP3 and MRP4 BA efflux activity.

### 4.3. FXR and Drug ADME

Experimental evidence supporting the protective role of FXR in DILI are growing. Nevertheless, rigorous drug-drug interactions studies are required before considering the pharmacological activation of FXR as an efficacious and feasible DILI risk-lowering strategy. In fact, several transporters and enzymes involved in BA and lipid metabolism that are regulated by FXR are polyspecific and play a major role also in the hepatic metabolism and clearance of several widely prescribed drugs, including APAP and valproic acid. If observed protective effects of FXR activation result from reduced intracellular levels of the perpetrator drug and/or its toxic metabolites, then the efficacy of the drug itself would be changed alongside its safety profile. Drug metabolism consists of four phases (0–3), each of which contribute to the overall plasmatic and/or hepatic drug levels and the resulting systemic and hepatic toxicity. Phase 0 describes the transport from the space of Disse into hepatocytes across the sinusoidal membrane, and is, for most drugs, mediated by organic anion transporting polypeptides (OATP) and by organic anion (OAT) and cation (OCT) transporters. Among others, OATP1B1 and OCT1 are downregulated by liganded FXR, whereas OATP1B3 expression level is induced by FXR activation [46,137]. Extensive genetic evidence demonstrates the pivotal role of OATP1B1, OATP1B3 and OCT1 in the pharmacokinetic/pharmacodynamic (PK/PD) inter-patient variability of several widely prescribed drugs, such as statins, metformin, methotrexate and fexofenadine [138,139,140,141,142,143,144]. Phase I and phase II reactions chemically modify the parent molecules to increase the water solubility, thereby facilitating the excretion in the bile. Phase I metabolism describes a series of redox and hydrolysis reactions meant to increase water solubility of lipophilic molecules. The most relevant enzymes involved in phase I metabolism belong to the CYP450 superfamily. The 18 CYP450 families are involved in the oxidation of BAs, lipids and hormones, with CYP1A2, CYP2C9, CYP2D6 and CYP3A4/5 being also largely involved in drug oxidation, accounting for the oxidation of more than 70% of marketed drugs [145]. Notably the expression of CYP3A4 and CYP2C9 is increased upon FXR activation [53,115]. Induction or inhibition of CYPs under polypharmacy are among the most frequent reasons for drug-drug interactions, resulting in altered PK/PD and drug toxicities. Phase II metabolism mainly consists in the conjugation of drugs and/or their metabolites with hydrophilic groups to further increase the polarity of the parent molecule. The most common conjugation reactions are glucoronidation and sulfation. UDP-glucuronosyltransferase enzymes (UGTs) catalyze the covalent addition of sugars to hydroxyl, carboxyl or amino groups [146]. It has been shown in human primary hepatocytes isolated from liver tissue and in HepG2 cells that UGT2B4 transcription is induced by FXR activation [147]. Phase III metabolism describes the elimination of drugs and metabolites from the hepatocyte in the canaliculi or back into the blood stream, a transport reaction primarily mediated by MRP2, localized on the canalicular membrane, and MRP3 and 4, localized on the sinusoidal membrane. FXR activation seems to induce the expression of MRP2 and 3, and to repress that of MRP4 [64,65,148]. Although clinical data correlating FXR expression and activation, and drug metabolism are missing, it is conceivable that the hepatic clearance of drugs whose hepatic metabolism involves transporters and enzymes under the regulation of FXR, might be affected, to some extent, by the expression and the activation state of FXR.

## 5. FXR Agonists—A Double-Edged Sword

Many high-affinity ligands for FXR are in development for the treatment of liver diseases [6], with a handful advancing into clinical trials [149,150,151,152,153,154]. In 2010, the semi-synthetic FXR agonist obeticholic acid (OCA) received the orphan drug status, and was approved for primary biliary cholangitis treatment in 2016 [155]. In 2018 a double-blinded, placebo-controlled phase II study evaluating the effect of OCA in patients with alcoholic hepatitis was terminated early [NCT02039219], as hepatotoxicity was documented in the post-marketing reports. Additional side effects were observed and described in the past years, such as atherogenesis and pruritus [153,156,157]. In 2021, the Food and Drug Agency submitted a Drug Safety Communication raising awareness that OCA treatment provoked severe hepatotoxicity in 25 patients with primary biliary cholangitis with advanced cirrhosis [158]. In parallel, the naturally occurring primary BA chenodeoxycholic acid, which is approved for treating a rare autosomal recessive disorder called cerebrotendinous xanthomatosis, has received a black box warning as hepatotoxic effects occurred in some instances [159,160]. With the purpose of further investigation and amelioration of OCA treatment, preclinical and clinical trials are being conducted to study the effects of OCA treatment in comparison or combination with other drugs. Recently, it was found that both OCA and Px-102, two structurally different FXR agonists, damage mitochondria, inducing oxidative stress both in vitro and in vivo (male Sprague–Dawley rats and male C57/bl6 mice) [161]. Additionally, it was shown in another study that high doses of OCA induced hepatotoxicity. The overall survival of wild-type but not FXR^-/-^ mice was reduced, indicating that the hepatotoxicity is not an off-target effect of OCA but stems from the constitutive activation of FXR [162]. A more recent clinical study confirmed that the treatment with OCA, especially when combined sequentially with ursodeoxycholic acid and fibrates, improves biochemical liver values and pruritus in patients with primary biliary cholangitis [160]. Nevertheless, the Food and Drug Agency considered re-evaluating to which extent OCA should be promoted or prohibited as a treatment for liver disease. Numerous FXR-based therapies underwent phase II or III clinical examinations to validate the safety and efficacy profiles when treating NASH, i.e., EDP305, tropifexor, cilofexor, nidufexor, TERN.101, Px-104, EYP001, MET409. Although all of these effectively reduced liver steatosis and fibrosis, class- and dose-related adverse events occurred. These adverse events are similar to those seen in response to OCA treatment, such as pruritus, elevated plasma levels of cholesterol and low-density lipoprotein, and diminished quantities of high-density lipoproteins.

## 6. Conclusions

We provided compelling evidence indicating that hepatic FXR preserves the integrity and function of hepatocytes under chronic and acute stress of varying nature. Yet, although not covered in the review article, it is not to be excluded that intestinal FXR also contributes to hepatoprotection by preserving the integrity of the intestinal mucosa and, in turn, sparing the liver from exposure to pathogens and toxins (e.g., secondary BAs). The myriad of signaling modalities of FXR, interconnected with pathways involved in inflammation and general cell function and health (autophagy, cellular energy metabolism), the existence of several isoforms in humans (and an additional gene *NR1H5* encoding FXRβ in other species), with differing signaling capacities, and the specific expression thereof across several tissues involved in BA and lipid homeostasis present a large complexity in the study and targeting of FXR for therapeutic purposes. While extensive preclinical studies have underscored the therapeutic potential of pharmacological activation of FXR in liver diseases, the clinical translation is hampered by unexpected side effects, including liver failure. Although not surprising, the discrepancy between animal and clinical studies remain unresolved and require further studies addressing the role of genetic predisposition and environmental factors, i.e., diet and microbiota, on the efficacy and safety of FXR agonists.

## Figures and Tables

**Table 1:** Effects of pharmacologic activation of FXR on drug-induced liver injury.

Compound	Etiopathogenesis of Liver Disease	Type of Study	Outcome	Ref.
GW4064	Acetaminophen-induced toxicity	Mouse	- Reduced serum ALT, AST and TNFα levels - Reduced liver necrosis	[126]
GW4064	Acetaminophen-induced toxicity	In vitro Mouse	- Upregulation of glutathione cycle genes - Reduced serum ALT, AST and LDH levels - Reduced liver necrosis	[130]
GW4064	Amoxicillin/Clavulanic acid-induced toxicity	In vitro	- GW4064 failed to activate FXR in cells treated with clavulanic acid.	[133]
Schaftoside	Acetaminophen-induced toxicity	Mouse	- Upregulation of glutathione cycle genes - Reduced serum, AST and LDH levels - Reduced hepatic oxidative stress and inflammation	[127]
Saffron	Acetaminophen-induced toxicity	Rat	- Reduced serum ALT, AST and LDH levels - Reduced hepatic glycogen depletion, sinusoidal dilation, vacuolization, bile stasis and necrosis	[128]
OCA	*Tripterygium*-induced toxicity	Mouse	- Reduced serum ALT, AST, ALP and TBA - Reduced histological alterations	[131]
OCA	Valproic acid-induced toxicity	In vitro Mouse	- Upregulation of glutathione cycle genes - Reduced hepatic oxidative stress - Reduced hepatic steatosis - Reduced hepatic oxida-tive stress - Upregulation of glutathione cycle genes - Downregulation of PPARγ gene	[132]

ALP = alkaline phosphatase, ALT = alanine aminotransferase, AST = aspartate transaminase, FXR = farnesoid X receptor, LDH = lactate dehydrogenase, PPARγ = peroxisome proliferator-activated receptor, OCA = Obeticholic acid, TBA = total bilirubin.

## Abbreviations

ABCG	ATP binding cassette subfamily G
ACC	Acetyl-CoA carboxylase
AMPK	AMP-activated protein kinase
APAP	Acetaminophen (Paracetamol)
BA	Bile acid
BSEP	Bile salt efflux pump
CD36	Cluster of differentiation 36; fatty acid translocase
CoA	Coenzyme A
CPT1	Carnitine palmitoyltransferase-1
CYP	Cytochrome P450 superfamily
DILI	Drug-induced liver injury
EET	Epoxyeicosatrienoic acid
EPHX2	Epoxide hydrolase 2
FA	Fatty acid
FABP	Fatty acid binding proteins
FAS	Fatty acid synthetase
FGF	Fibroblast growth factor
FXR	Farnesoid X receptor
HNF4α	Hepatocyte nuclear factor 4 alpha
LDLr	Low-density lipoprotein receptor
LRH-1	Liver receptor homolog-1
LTB	Leukotriene
MDR3	Multidrug resistance protein 3
MRP	Multidrug resistance associated protein
NAFLD	Nonalcoholic fatty liver disease
NASH	Nonalcoholic steatohepatits
NF-κB	Nuclear factor kappa B
NTCP	Sodium-taurocholate cotransporting polypeptide
OAT	Organic anion transporter
OATP	Organic anion transporting polypeptides
OCA	Obeticholic acid
OCT	Organic cation transporter
OSTα/β	Organic solute transporter alpha/beta
PPAR	Peroxisome proliferator-activated receptor
RXR	Retinoid X receptor
SHP-1	Small heterodimer protein 1
SIRT1	Sirtuin 1; class III NAD+ dependent histone deacetylase
SREBP-1c	Sterol regulatory element-binding protein 1c
UGT	UDP-glucuronosyltransferase enzymes
VLDL	Very-low density lipoprotein

## References

[1] Scholtes C., Giguère V. (2022). Transcriptional control of energy metabolism by nuclear receptors. Nat. Rev. Mol. Cell Biol..

[2] Achermann J.C., Schwabe J., Fairall L., Chatterjee K. (2017). Genetic disorders of nuclear receptors. J. Clin. Investig..

[3] Wagner M., Zollner G., Trauner M. (2011). Nuclear receptors in liver disease. Hepatology.

[4] Cave M.C., Clair H.B., Hardesty J.E., Falkner K.C., Feng W., Clark B.J., Sidey J., Shi H., Aqel B.A., McClain C.J. (2016). Nuclear receptors and nonalcoholic fatty liver disease. Biochim. Biophys. Acta.

[5] Halilbasic E., Baghdasaryan A., Trauner M. (2013). Nuclear receptors as drug targets in cholestatic liver diseases. Clin. Liver Dis..

[6] Trauner M., Fuchs C.D. (2022). Novel therapeutic targets for cholestatic and fatty liver disease. Gut.

[7] Zhang Y., Kast-Woelbern H.R., Edwards P.A. (2003). Natural structural variants of the nuclear receptor farnesoid X receptor affect transcriptional activation. J. Biol. Chem..

[8] Ramos Pittol J.M., Milona A., Morris I., Willemsen E.C.L., van der Veen S.W., Kalkhoven E., van Mil S.W.C. (2020). FXR Isoforms Control Different Metabolic Functions in Liver Cells via Binding to Specific DNA Motifs. Gastroenterology.

[9] Vaquero J., Monte M.J., Dominguez M., Muntané J., Marin J.J.G. (2013). Differential activation of the human farnesoid X receptor depends on the pattern of expressed isoforms and the bile acid pool composition. Biochem. Pharmacol..

[10] Jiang L., Zhang H., Xiao D., Wei H., Chen Y. (2021). Farnesoid X receptor (FXR): Structures and ligands. Comput. Struct. Biotechnol. J..

[11] Song X., Chen Y., Valanejad L., Kaimal R., Yan B., Stoner M., Deng R. (2013). Mechanistic insights into isoform-dependent and species-specific regulation of bile salt export pump by farnesoid X receptor. J. Lipid Res..

[12] Huber R.M., Murphy K., Miao B., Link J.R., Cunningham M.R., Rupar M.J., Gunyuzlu P.L., Haws T.F., Kassam A., Powell F. (2002). Generation of multiple farnesoid-X-receptor isoforms through the use of alternative promoters. Gene.

[13] Mustonen E.K., Lee S.M.L., Nieß H., Schwab M., Pantsar T., Burk O. (2021). Identification and characterization of novel splice variants of human farnesoid X receptor. Arch. Biochem. Biophys..

[14] Forman B.M., Goode E., Chen J., Oro A.E., Bradley D.J., Perlmann T., Noonan D.J., Burka L.T., McMorris T., Lamph W.W. (1995). Identification of a nuclear receptor that is activated by farnesol metabolites. Cell.

[15] Wang H., Chen J., Hollister K., Sowers L.C., Forman B.M. (1999). Endogenous Bile Acids Are Ligands for the Nuclear Receptor FXR/BAR. Mol. Cell.

[16] Makishima M., Okamoto A.Y., Repa J.J., Tu H., Learned R.M., Luk A., Hull M.V., Lustig K.D., Mangelsdorf D.J., Shan B. (1999). Identification of a nuclear receptor for bile acids. Science.

[17] Mi L.-Z., Devarakonda S., Harp J.M., Han Q., Pellicciari R., Willson T.M., Khorasanizadeh S., Rastinejad F. (2003). Structural Basis for Bile Acid Binding and Activation of the Nuclear Receptor FXR. Mol. Cell.

[18] Otte K., Kranz H., Kober I., Thompson P., Hoefer M., Haubold B., Remmel B., Voss H., Kaiser C., Albers M. (2003). Identification of farnesoid X receptor beta as a novel mammalian nuclear receptor sensing lanosterol. Mol. Cell. Biol..

[19] Wang S., Lai K., Moy F.J., Bhat A., Hartman H.B., Evans M.J. (2006). The Nuclear Hormone Receptor Farnesoid X Receptor (FXR) Is Activated by Androsterone. Endocrinology.

[20] Howard W.R., Pospisil J.A., Njolito E., Noonan D.J. (2000). Catabolites of cholesterol synthesis pathways and forskolin as activators of the farnesoid X-activated nuclear receptor. Toxicol. Appl. Pharmacol..

[21] Ricketts M.L., Boekschoten M.V., Kreeft A.J., Hooiveld G.J., Moen C.J., Müller M., Frants R.R., Kasanmoentalib S., Post S.M., Princen H.M. (2007). The cholesterol-raising factor from coffee beans, cafestol, as an agonist ligand for the farnesoid and pregnane X receptors. Mol. Endocrinol..

[22] Vacca M., Degirolamo C., Mariani-Costantini R., Palasciano G., Moschetta A. (2011). Lipid-sensing nuclear receptors in the pathophysiology and treatment of the metabolic syndrome. WIREs Syst. Biol. Med..

[23] Laffitte B.A., Kast H.R., Nguyen C.M., Zavacki A.M., Moore D.D., Edwards P.A. (2000). Identification of the DNA Binding Specificity and Potential Target Genes for the Farnesoid X-activated Receptor*. J. Biol. Chem..

[24] Claudel T., Sturm E., Duez H., Torra I.P., Sirvent A., Kosykh V., Fruchart J.C., Dallongeville J., Hum D.W., Kuipers F. (2002). Bile acid-activated nuclear receptor FXR suppresses apolipoprotein A-I transcription via a negative FXR response element. J. Clin. Investig..

[25] Xu X., Xu X., Liu P., Zhu Z.Y., Chen J., Fu H.A., Chen L.L., Hu L.H., Shen X. (2015). Structural Basis for Small Molecule NDB (N-Benzyl-N-(3-(tert-butyl)-4-hydroxyphenyl)-2,6-dichloro-4-(dimethylamino) Benzamide) as a Selective Antagonist of Farnesoid X Receptor α (FXRα) in Stabilizing the Homodimerization of the Receptor. J. Biol. Chem..

[26] Kemper J.K. (2011). Regulation of FXR transcriptional activity in health and disease: Emerging roles of FXR cofactors and post-translational modifications. Biochim. Biophys. Acta..

[27] Chong H.K., Infante A.M., Seo Y.K., Jeon T.I., Zhang Y., Edwards P.A., Xie X., Osborne T.F. (2010). Genome-wide interrogation of hepatic FXR reveals an asymmetric IR-1 motif and synergy with LRH-1. Nucleic Acids Res..

[28] Armstrong L.E., Guo G.L. (2017). Role of FXR in Liver Inflammation during Nonalcoholic Steatohepatitis. Curr. Pharmacol. Rep..

[29] Zhu Y., Liu H., Zhang M., Guo G.L. (2016). Fatty liver diseases, bile acids, and FXR. Acta Pharm. Sin. B.

[30] Carr R.M., Reid A.E. (2015). FXR Agonists as Therapeutic Agents for Non-alcoholic Fatty Liver Disease. Curr. Atheroscler. Rep..

[31] Stofan M., Guo G.L. (2020). Bile Acids and FXR: Novel Targets for Liver Diseases. Front. Med..

[32] Hofmann A.F. (2009). Bile acids: Trying to understand their chemistry and biology with the hope of helping patients. Hepatology.

[33] Russell D.W. (2003). The enzymes, regulation, and genetics of bile acid synthesis. Annu. Rev. Biochem..

[34] Pillot T., Ouzzine M., Fournel-Gigleux S., Lafaurie C., Radominska A., Burchell B., Siest G., Magdalou J. (1993). Glucuronidation of hyodeoxycholic acid in human liver. Evidence for a selective role of UDP-glucuronosyltransferase 2B4. J. Biol. Chem..

[35] Han J., Liu Y., Wang R., Yang J., Ling V., Borchers C.H. (2015). Metabolic profiling of bile acids in human and mouse blood by LC-MS/MS in combination with phospholipid-depletion solid-phase extraction. Anal. Chem..

[36] Trottier J., Verreault M., Grepper S., Monté D., Bélanger J., Kaeding J., Caron P., Inaba T.T., Barbier O. (2006). Human UDP-glucuronosyltransferase (UGT)1A3 enzyme conjugates chenodeoxycholic acid in the liver. Hepatology.

[37] Stieger B., Meier Y., Meier P.J. (2007). The bile salt export pump. Pflug. Arch..

[38] Soroka C.J., Lee J.M., Azzaroli F., Boyer J.L. (2001). Cellular localization and up-regulation of multidrug resistance-associated protein 3 in hepatocytes and cholangiocytes during obstructive cholestasis in rat liver. Hepatology.

[39] Denk G.U., Soroka C.J., Takeyama Y., Chen W.S., Schuetz J.D., Boyer J.L. (2004). Multidrug resistance-associated protein 4 is up-regulated in liver but down-regulated in kidney in obstructive cholestasis in the rat. J. Hepatol..

[40] Donner M.G., Keppler D. (2001). Up-regulation of basolateral multidrug resistance protein 3 (Mrp3) in cholestatic rat liver. Hepatology.

[41] Landrier J.F., Eloranta J.J., Vavricka S.R., Kullak-Ublick G.A. (2006). The nuclear receptor for bile acids, FXR, transactivates human organic solute transporter-α and -β genes. Am. J. Physiol.-Gastrointest. Liver Physiol..

[42] Ballatori N., Christian W.V., Lee J.Y., Dawson P.A., Soroka C.J., Boyer J.L., Madejczyk M.S., Li N. (2005). OSTα-OSTβ: A major basolateral bile acid and steroid transporter in human intestinal, renal, and biliary epithelia. Hepatology.

[43] Chen J., Zhao K.N., Chen C. (2014). The role of CYP3A4 in the biotransformation of bile acids and therapeutic implication for cholestasis. Ann. Transl. Med..

[44] Ridlon J.M., Kang D.J., Hylemon P.B. (2006). Bile salt biotransformations by human intestinal bacteria. J. Lipid Res..

[45] Hofmann A.F. (2009). The enterohepatic circulation of bile acids in mammals: Form and functions. Front. Biosci. Landmark Ed..

[46] Kullak-Ublick G.A., Stieger B., Meier P.J. (2004). Enterohepatic bile salt transporters in normal physiology and liver disease. Gastroenterology.

[47] Trauner M., Fickert P., Halilbasic E., Moustafa T. (2008). Lessons from the toxic bile concept for the pathogenesis and treatment of cholestatic liver diseases. Wien. Med. Wochenschr..

[48] Davit-Spraul A., Gonzales E., Baussan C., Jacquemin E. (2009). Progressive familial intrahepatic cholestasis. Orphanet J. Rare Dis..

[49] Wang L., Soroka C.J., Boyer J.L. (2002). The role of bile salt export pump mutations in progressive familial intrahepatic cholestasis type II. J. Clin. Investig..

[50] Rudkowska I., Jones P.J. (2008). Polymorphisms in ABCG5/G8 transporters linked to hypercholesterolemia and gallstone disease. Nutr. Rev..

[51] Vaz F.M., Ferdinandusse S. (2017). Bile acid analysis in human disorders of bile acid biosynthesis. Mol. Asp. Med..

[52] Chiang J.Y.L., Ferrell J.M. (2019). Bile Acids as Metabolic Regulators and Nutrient Sensors. Annu. Rev. Nutr..

[53] Gnerre C., Blättler S., Kaufmann M.R., Looser R., Meyer U.A. (2004). Regulation of CYP3A4 by the bile acid receptor FXR: Evidence for functional binding sites in the CYP3A4 gene. Pharmacogenetics.

[54] Ananthanarayanan M., Balasubramanian N., Makishima M., Mangelsdorf D.J., Suchy F.J. (2001). Human Bile Salt Export Pump Promoter Is Transactivated by the Farnesoid X Receptor/Bile Acid Receptor*. J. Biol. Chem..

[55] Eloranta J.J., Jung D., Kullak-Ublick G.A. (2006). The human Na^+^-taurocholate cotransporting polypeptide gene is activated by glucocorticoid receptor and peroxisome proliferator-activated receptor-γ coactivator-1α, and suppressed by bile acids via a small heterodimer partner-dependent mechanism. Mol. Endocrinol..

[56] Goodwin B., Jones S.A., Price R.R., Watson M.A., McKee D.D., Moore L.B., Galardi C., Wilson J.G., Lewis M.C., Roth M.E. (2000). A regulatory cascade of the nuclear receptors FXR, SHP-1, and LRH-1 represses bile acid biosynthesis. Mol. Cell.

[57] Zhang M., Chiang J.Y. (2001). Transcriptional regulation of the human sterol 12α-hydroxylase gene (CYP8B1): Roles of heaptocyte nuclear factor 4α in mediating bile acid repression. J. Biol. Chem..

[58] Jung D., Kullak-Ublick G.A. (2003). Hepatocyte nuclear factor 1 α: A key mediator of the effect of bile acids on gene expression. Hepatology.

[59] Inagaki T., Choi M., Moschetta A., Peng L., Cummins C.L., McDonald J.G., Luo G., Jones S.A., Goodwin B., Richardson J.A. (2005). Fibroblast growth factor 15 functions as an enterohepatic signal to regulate bile acid homeostasis. Cell Metab..

[60] Huang L., Zhao A., Lew J.L., Zhang T., Hrywna Y., Thompson J.R., de Pedro N., Royo I., Blevins R.A., Peláez F. (2003). Farnesoid X receptor activates transcription of the phospholipid pump MDR3. J. Biol. Chem..

[61] Li T., Matozel M., Boehme S., Kong B., Nilsson L.M., Guo G., Ellis E., Chiang J.Y. (2011). Overexpression of cholesterol 7α-hydroxylase promotes hepatic bile acid synthesis and secretion and maintains cholesterol homeostasis. Hepatology.

[62] Gomez-Ospina N., Potter C.J., Xiao R., Manickam K., Kim M.S., Kim K.H., Shneider B.L., Picarsic J.L., Jacobson T.A., Zhang J. (2016). Mutations in the nuclear bile acid receptor FXR cause progressive familial intrahepatic cholestasis. Nat. Commun..

[63] Van Mil S.W., Milona A., Dixon P.H., Mullenbach R., Geenes V.L., Chambers J., Shevchuk V., Moore G.E., Lammert F., Glantz A.G. (2007). Functional variants of the central bile acid sensor FXR identified in intrahepatic cholestasis of pregnancy. Gastroenterology.

[64] Stedman C., Liddle C., Coulter S., Sonoda J., Alvarez J.G., Evans R.M., Downes M. (2006). Benefit of farnesoid X receptor inhibition in obstructive cholestasis. Proc. Natl. Acad. Sci. USA.

[65] Renga B., Migliorati M., Mencarelli A., Cipriani S., D’Amore C., Distrutti E., Fiorucci S. (2011). Farnesoid X receptor suppresses constitutive androstane receptor activity at the multidrug resistance protein-4 promoter. Biochim. Biophys. Acta.

[66] Abumrad N., Coburn C., Ibrahimi A. (1999). Membrane proteins implicated in long-chain fatty acid uptake by mammalian cells: CD36, FATP and FABPm. Biochim. Biophys. Acta.

[67] Chakravarty B., Gu Z., Chirala S.S., Wakil S.J., Quiocho F.A. (2004). Human fatty acid synthase: Structure and substrate selectivity of the thioesterase domain. Proc. Natl. Acad. Sci. USA.

[68] Houten S.M., Wanders R.J. (2010). A general introduction to the biochemistry of mitochondrial fatty acid β-oxidation. J. Inherit. Metab. Dis..

[69] Bartlett K., Eaton S. (2004). Mitochondrial beta-oxidation. Eur. J. Biochem..

[70] Fan C.Y., Pan J., Usuda N., Yeldandi A.V., Rao M.S., Reddy J.K. (1998). Steatohepatitis, spontaneous peroxisome proliferation and liver tumors in mice lacking peroxisomal fatty acyl-CoA oxidase. Implications for peroxisome proliferator-activated receptor α natural ligand metabolism. J. Biol. Chem..

[71] Wanders R.J., Komen J., Kemp S. (2011). Fatty acid omega-oxidation as a rescue pathway for fatty acid oxidation disorders in humans. FEBS J..

[72] Vance J.E. (2015). Phospholipid synthesis and transport in mammalian cells. Traffic.

[73] Hanada K. (2003). Serine palmitoyltransferase, a key enzyme of sphingolipid metabolism. Biochim. Biophys. Acta.

[74] Brunt E.M., Wong V.W., Nobili V., Day C.P., Sookoian S., Maher J.J., Bugianesi E., Sirlin C.B., Neuschwander-Tetri B.A., Rinella M.E. (2015). Nonalcoholic fatty liver disease. Nat. Rev. Dis. Prim..

[75] Sookoian S., Pirola C.J. (2017). Genetic predisposition in nonalcoholic fatty liver disease. Clin. Mol. Hepatol..

[76] Galic S., Loh K., Murray-Segal L., Steinberg G.R., Andrews Z.B., Kemp B.E. (2018). AMPK signaling to acetyl-CoA carboxylase is required for fasting- and cold-induced appetite but not thermogenesis. eLife.

[77] Smith B.K., Marcinko K., Desjardins E.M., Lally J.S., Ford R.J., Steinberg G.R. (2016). Treatment of nonalcoholic fatty liver disease: Role of AMPK. Am. J. Physiol. Endocrinol. Metab..

[78] Liu M., Alimov A.P., Wang H., Frank J.A., Katz W., Xu M., Ke Z.J., Luo J. (2014). Thiamine deficiency induces anorexia by inhibiting hypothalamic AMPK. Neuroscience.

[79] Chen L., Shu Y., Liang X., Chen E.C., Yee S.W., Zur A.A., Li S., Xu L., Keshari K.R., Lin M.J. (2014). OCT1 is a high-capacity thiamine transporter that regulates hepatic steatosis and is a target of metformin. Proc. Natl. Acad. Sci. USA.

[80] Rada P., González-Rodríguez Á., García-Monzón C., Valverde Á.M. (2020). Understanding lipotoxicity in NAFLD pathogenesis: Is CD36 a key driver?. Cell Death Dis..

[81] Huang F., Wang T., Lan Y., Yang L., Pan W., Zhu Y., Lv B., Wei Y., Shi H., Wu H. (2015). Deletion of mouse FXR gene disturbs multiple neurotransmitter systems and alters neurobehavior. Front. Behav. Neurosci..

[82] Prawitt J., Abdelkarim M., Stroeve J.H., Popescu I., Duez H., Velagapudi V.R., Dumont J., Bouchaert E., van Dijk T.H., Lucas A. (2011). Farnesoid X receptor deficiency improves glucose homeostasis in mouse models of obesity. Diabetes.

[83] Bjursell M., Wedin M., Admyre T., Hermansson M., Böttcher G., Göransson M., Lindén D., Bamberg K., Oscarsson J., Bohlooly Y.M. (2013). Ageing Fxr deficient mice develop increased energy expenditure, improved glucose control and liver damage resembling NASH. PLoS ONE.

[84] Sinal C.J., Tohkin M., Miyata M., Ward J.M., Lambert G., Gonzalez F.J. (2000). Targeted disruption of the nuclear receptor FXR/BAR impairs bile acid and lipid homeostasis. Cell.

[85] Saborowski M., Kullak-Ublick G.A., Eloranta J.J. (2006). The human organic cation transporter-1 gene is transactivated by hepatocyte nuclear factor-4α. J. Pharmacol. Exp. Ther..

[86] Watanabe M., Houten S.M., Wang L., Moschetta A., Mangelsdorf D.J., Heyman R.A., Moore D.D., Auwerx J. (2004). Bile acids lower triglyceride levels via a pathway involving FXR, SHP, and SREBP-1c. J. Clin. Investig..

[87] Clifford B.L., Sedgeman L.R., Williams K.J., Morand P., Cheng A., Jarrett K.E., Chan A.P., Brearley-Sholto M.C., Wahlström A., Ashby J.W. (2021). FXR activation protects against NAFLD via bile-acid-dependent reductions in lipid absorption. Cell Metab..

[88] Nakayama H., Otabe S., Ueno T., Hirota N., Yuan X., Fukutani T., Hashinaga T., Wada N., Yamada K. (2007). Transgenic mice expressing nuclear sterol regulatory element-binding protein 1c in adipose tissue exhibit liver histology similar to nonalcoholic steatohepatitis. Metabolism.

[89] Foufelle F., Ferré P. (2002). New perspectives in the regulation of hepatic glycolytic and lipogenic genes by insulin and glucose: A role for the transcription factor sterol regulatory element binding protein-1c. Biochem. J..

[90] Pettinelli P., Videla L.A. (2011). Up-regulation of PPAR-gamma mRNA expression in the liver of obese patients: An additional reinforcing lipogenic mechanism to SREBP-1c induction. J. Clin. Endocrinol. Metab..

[91] Yang X., Zhang W., Chen Y., Li Y., Sun L., Liu Y., Liu M., Yu M., Li X., Han J. (2016). Activation of Peroxisome Proliferator-activated Receptor γ (PPARγ) and CD36 Protein Expression: The Dual Pathophysiological Roles of Progesterone. J. Biol. Chem..

[92] Gavrilova O., Haluzik M., Matsusue K., Cutson J.J., Johnson L., Dietz K.R., Nicol C.J., Vinson C., Gonzalez F.J., Reitman M.L. (2003). Liver peroxisome proliferator-activated receptor gamma contributes to hepatic steatosis, triglyceride clearance, and regulation of body fat mass. J. Biol. Chem..

[93] Matsusue K., Haluzik M., Lambert G., Yim S.H., Gavrilova O., Ward J.M., Brewer B., Reitman M.L., Gonzalez F.J. (2003). Liver-specific disruption of PPARgamma in leptin-deficient mice improves fatty liver but aggravates diabetic phenotypes. J. Clin. Investig..

[94] Zhang W., Sun Q., Zhong W., Sun X., Zhou Z. (2016). Hepatic Peroxisome Proliferator-Activated Receptor Gamma Signaling Contributes to Alcohol-Induced Hepatic Steatosis and Inflammation in Mice. Alcohol. Clin. Exp. Res..

[95] Gai Z., Gui T., Hiller C., Kullak-Ublick G.A. (2016). Farnesoid X Receptor Protects against Kidney Injury in Uninephrectomized Obese Mice. J. Biol. Chem..

[96] Du J., Xiang X., Xu D., Zhang J., Fang W., Xu W., Mai K., Ai Q. (2021). FXR, a Key Regulator of Lipid Metabolism, Is Inhibited by ER Stress-Mediated Activation of JNK and p38 MAPK in Large Yellow Croakers (*Larimichthys crocea*) Fed High Fat Diets. Nutrients.

[97] Correia J.C., Massart J., de Boer J.F., Porsmyr-Palmertz M., Martínez-Redondo V., Agudelo L.Z., Sinha I., Meierhofer D., Ribeiro V., Björnholm M. (2015). Bioenergetic cues shift FXR splicing towards FXRα2 to modulate hepatic lipolysis and fatty acid metabolism. Mol. Metab..

[98] Pineda Torra I., Claudel T., Duval C., Kosykh V., Fruchart J.C., Staels B. (2003). Bile acids induce the expression of the human peroxisome proliferator-activated receptor alpha gene via activation of the farnesoid X receptor. Mol. Endocrinol..

[99] Zhou S., You H., Qiu S., Yu D., Bai Y., He J., Cao H., Che Q., Guo J., Su Z. (2022). A new perspective on NAFLD: Focusing on the crosstalk between peroxisome proliferator-activated receptor alpha (PPARα) and farnesoid X receptor (FXR). Biomed. Pharmacother..

[100] Zhang Y., Lickteig A.J., Csanaky I.L., Klaassen C.D. (2017). Editor’s Highlight: Clofibrate Decreases Bile Acids in Livers of Male Mice by Increasing Biliary Bile Acid Excretion in a PPARα-Dependent Manner. Toxicol. Sci..

[101] Cariello M., Piccinin E., Moschetta A. (2021). Transcriptional Regulation of Metabolic Pathways via Lipid-Sensing Nuclear Receptors PPARs, FXR, and LXR in NASH. Cell. Mol. Gastroenterol. Hepatol..

[102] Lee J.M., Wagner M., Xiao R., Kim K.H., Feng D., Lazar M.A., Moore D.D. (2014). Nutrient-sensing nuclear receptors coordinate autophagy. Nature.

[103] Mazzini G.S., Khoraki J., Browning M.G., Wu J., Zhou H., Price E.T., Wolfe L.G., Mangino M.J., Campos G.M. (2021). Gastric Bypass Increases Circulating Bile Acids and Activates Hepatic Farnesoid X Receptor (FXR) but Requires Intact Peroxisome Proliferator Activator Receptor Alpha (PPARα) Signaling to Significantly Reduce Liver Fat Content. J. Gastrointest. Surg..

[104] Othman A., Saely C.H., Muendlein A., Vonbank A., Drexel H., von Eckardstein A., Hornemann T. (2015). Plasma 1-deoxysphingolipids are predictive biomarkers for type 2 diabetes mellitus. BMJ Open Diabetes Res. Care.

[105] Gai Z., Gui T., Alecu I., Lone M.A., Hornemann T., Chen Q., Visentin M., Hiller C., Hausler S., Kullak-Ublick G.A. (2020). Farnesoid X receptor activation induces the degradation of hepatotoxic 1-deoxysphingolipids in non-alcoholic fatty liver disease. Liver Int..

[106] Alecu I., Tedeschi A., Behler N., Wunderling K., Lamberz C., Lauterbach M.A., Gaebler A., Ernst D., van Veldhoven P.P., Al-Amoudi A. (2017). Localization of 1-deoxysphingolipids to mitochondria induces mitochondrial dysfunction. J. Lipid Res..

[107] Bettigole S.E., Glimcher L.H. (2015). Endoplasmic reticulum stress in immunity. Annu. Rev. Immunol..

[108] Alecu I., Othman A., Penno A., Saied E.M., Arenz C., von Eckardstein A., Hornemann T. (2017). Cytotoxic 1-deoxysphingolipids are metabolized by a cytochrome P450-dependent pathway. J. Lipid Res..

[109] Kong B., Luyendyk J.P., Tawfik O., Guo G.L. (2009). Farnesoid X receptor deficiency induces nonalcoholic steatohepatitis in low-density lipoprotein receptor-knockout mice fed a high-fat diet. J. Pharmacol. Exp. Ther..

[110] Wang Y.D., Chen W.D., Wang M., Yu D., Forman B.M., Huang W. (2008). Farnesoid X receptor antagonizes nuclear factor kappaB in hepatic inflammatory response. Hepatology.

[111] Yao J., Zhou C.-S., Ma X., Fu B.-Q., Tao L.-S., Chen M., Xu Y.-P. (2014). FXR agonist GW4064 alleviates endotoxin-induced hepatic inflammation by repressing macrophage activation. World J. Gastroenterol..

[112] Miyazaki-Anzai S., Levi M., Kratzer A., Ting T.C., Lewis L.B., Miyazaki M. (2010). Farnesoid X receptor activation prevents the development of vascular calcification in ApoE^−/−^ mice with chronic kidney disease. Circ. Res..

[113] Hu Z., Ren L., Wang C., Liu B., Song G. (2012). Effect of chenodeoxycholic acid on fibrosis, inflammation and oxidative stress in kidney in high-fructose-fed Wistar rats. Kidney Blood Press. Res..

[114] Jiang T., Wang X.X., Scherzer P., Wilson P., Tallman J., Takahashi H., Li J., Iwahashi M., Sutherland E., Arend L. (2007). Farnesoid X receptor modulates renal lipid metabolism, fibrosis, and diabetic nephropathy. Diabetes.

[115] Gai Z., Visentin M., Gui T., Zhao L., Thasler W.E., Häusler S., Hartling I., Cremonesi A., Hiller C., Kullak-Ublick G.A. (2018). Effects of Farnesoid X Receptor Activation on Arachidonic Acid Metabolism, NF-kB Signaling, and Hepatic Inflammation. Mol. Pharmacol..

[116] Kim D.-H., Xiao Z., Kwon S., Sun X., Ryerson D., Tkac D., Ma P., Wu S.-Y., Chiang C.-M., Zhou E. (2015). A dysregulated acetyl/SUMO switch of FXR promotes hepatic inflammation in obesity. EMBO J..

[117] Pols T.W., Noriega L.G., Nomura M., Auwerx J., Schoonjans K. (2011). The bile acid membrane receptor TGR5: A valuable metabolic target. Dig. Dis..

[118] Schote A.B., Turner J.D., Schiltz J., Muller C.P. (2007). Nuclear receptors in human immune cells: Expression and correlations. Mol. Immunol..

[119] Meadows V., Kennedy L., Ekser B., Kyritsi K., Kundu D., Zhou T., Chen L., Pham L., Wu N., Demieville J. (2021). Mast Cells Regulate Ductular Reaction and Intestinal Inflammation in Cholestasis Through Farnesoid X Receptor Signaling. Hepatology.

[120] Weston C.J., Zimmermann H.W., Adams D.H. (2019). The Role of Myeloid-Derived Cells in the Progression of Liver Disease. Front. Immunol..

[121] Fiorucci S., Biagioli M., Sepe V., Zampella A., Distrutti E. (2020). Bile acid modulators for the treatment of nonalcoholic steatohepatitis (NASH). Expert Opin. Investig. Drugs.

[122] Pathak P., Liu H., Boehme S., Xie C., Krausz K.W., Gonzalez F., Chiang J.Y.L. (2017). Farnesoid X receptor induces Takeda G-protein receptor 5 cross-talk to regulate bile acid synthesis and hepatic metabolism. J. Biol. Chem..

[123] Fiorucci S., Zampella A., Ricci P., Distrutti E., Biagioli M. (2022). Immunomodulatory functions of FXR. Mol. Cell. Endocrinol..

[124] Visentin M., Lenggenhager D., Gai Z., Kullak-Ublick G.A. (2018). Drug-induced bile duct injury. Biochim. Biophys. Acta Mol. Basis Dis..

[125] Kullak-Ublick G.A., Andrade R.J., Merz M., End P., Benesic A., Gerbes A.L., Aithal G.P. (2017). Drug-induced liver injury: Recent advances in diagnosis and risk assessment. Gut.

[126] Yan T., Yan N., Wang H., Yagai T., Luo Y., Takahashi S., Zhao M., Krausz K.W., Wang G., Hao H. (2021). FXR-Deoxycholic Acid-TNF-α Axis Modulates Acetaminophen-Induced Hepatotoxicity. Toxicol. Sci..

[127] Liu M., Zhang G., Song M., Wang J., Shen C., Chen Z., Huang X., Gao Y., Zhu C., Lin C. (2020). Activation of Farnesoid X Receptor by Schaftoside Ameliorates Acetaminophen-Induced Hepatotoxicity by Modulating Oxidative Stress and Inflammation. Antioxid. Redox Signal..

[128] Jamshidi V., Hashemi S.A., Khalili A., Fallah P., Ahmadian-Attari M.M., Beikzadeh L., Mazloom R., Najafizadeh P., Bayat G. (2021). Saffron offers hepatoprotection via up-regulation of hepatic farnesoid-X-activated receptors in a rat model of acetaminophen-induced hepatotoxicity. Avicenna J. Phytomed..

[129] Kemper J.K., Xiao Z., Ponugoti B., Miao J., Fang S., Kanamaluru D., Tsang S., Wu S.Y., Chiang C.M., Veenstra T.D. (2009). FXR acetylation is normally dynamically regulated by p300 and SIRT1 but constitutively elevated in metabolic disease states. Cell Metab..

[130] Lee F.Y., de Aguiar Vallim T.Q., Chong H.K., Zhang Y., Liu Y., Jones S.A., Osborne T.F., Edwards P.A. (2010). Activation of the farnesoid X receptor provides protection against acetaminophen-induced hepatic toxicity. Mol. Endocrinol..

[131] Peng W., Dai M.Y., Bao L.J., Zhu W.F., Li F. (2021). FXR activation prevents liver injury induced by *Tripterygium wilfordii* preparations. Xenobiotica.

[132] Gai Z., Krajnc E., Samodelov S.L., Visentin M., Kullak-Ublick G.A. (2020). Obeticholic Acid Ameliorates Valproic Acid-Induced Hepatic Steatosis and Oxidative Stress. Mol. Pharmacol..

[133] Petrov P.D., Soluyanova P., Sánchez-Campos S., Castell J.V., Jover R. (2021). Molecular mechanisms of hepatotoxic cholestasis by clavulanic acid: Role of NRF2 and FXR pathways. Food Chem. Toxicol..

[134] Padda M.S., Sanchez M., Akhtar A.J., Boyer J.L. (2011). Drug-induced cholestasis. Hepatology.

[135] Sundaram V., Björnsson E.S. (2017). Drug-induced cholestasis. Hepatol. Commun..

[136] Mahdi Z.M., Synal-Hermanns U., Yoker A., Locher K.P., Stieger B. (2016). Role of Multidrug Resistance Protein 3 in Antifungal-Induced Cholestasis. Mol. Pharmacol..

[137] Jung D., Podvinec M., Meyer U.A., Mangelsdorf D.J., Fried M., Meier P.J., Kullak-Ublick G.A. (2002). Human organic anion transporting polypeptide 8 promoter is transactivated by the farnesoid X receptor/bile acid receptor. Gastroenterology.

[138] Link E., Parish S., Armitage J., Bowman L., Heath S., Matsuda F., Gut I., Lathrop M., Collins R. (2008). SLCO1B1 variants and statin-induced myopathy—A genomewide study. N. Engl. J. Med..

[139] Kameyama Y., Yamashita K., Kobayashi K., Hosokawa M., Chiba K. (2005). Functional characterization of SLCO1B1 (OATP-C) variants, SLCO1B1*5, SLCO1B1*15 and SLCO1B1*15+C1007G, by using transient expression systems of HeLa and HEK293 cells. Pharm. Genom..

[140] Trevino L.R., Shimasaki N., Yang W., Panetta J.C., Cheng C., Pei D., Chan D., Sparreboom A., Giacomini K.M., Pui C.H. (2009). Germline genetic variation in an organic anion transporter polypeptide associated with methotrexate pharmacokinetics and clinical effects. J. Clin. Oncol..

[141] Ramsey L.B., Panetta J.C., Smith C., Yang W., Fan Y., Winick N.J., Martin P.L., Cheng C., Devidas M., Pui C.H. (2013). Genome-wide study of methotrexate clearance replicates SLCO1B1. Blood.

[142] Niemi M., Kivisto K.T., Hofmann U., Schwab M., Eichelbaum M., Fromm M.F. (2005). Fexofenadine pharmacokinetics are associated with a polymorphism of the SLCO1B1 gene (encoding OATP1B1). Br. J. Clin. Pharmacol..

[143] Pasanen M.K., Fredrikson H., Neuvonen P.J., Niemi M. (2007). Different effects of SLCO1B1 polymorphism on the pharmacokinetics of atorvastatin and rosuvastatin. Clin. Pharmacol. Ther..

[144] Kohlrausch F.B., de Cassia Estrela R., Barroso P.F., Suarez-Kurtz G. (2010). The impact of SLCO1B1 polymorphisms on the plasma concentration of lopinavir and ritonavir in HIV-infected men. Br. J. Clin. Pharmacol..

[145] Esteves F., Rueff J., Kranendonk M. (2021). The Central Role of Cytochrome P450 in Xenobiotic Metabolism—A Brief Review on a Fascinating Enzyme Family. J. Xenobiot..

[146] Guillemette C., Lévesque É., Rouleau M. (2014). Pharmacogenomics of human uridine diphospho-glucuronosyltransferases and clinical implications. Clin. Pharmacol. Ther..

[147] Barbier O., Torra I.P., Sirvent A., Claudel T., Blanquart C., Duran-Sandoval D., Kuipers F., Kosykh V., Fruchart J.C., Staels B. (2003). FXR induces the UGT2B4 enzyme in hepatocytes: A potential mechanism of negative feedback control of FXR activity. Gastroenterology.

[148] Zollner G., Fickert P., Fuchsbichler A., Silbert D., Wagner M., Arbeiter S., Gonzalez F.J., Marschall H.U., Zatloukal K., Denk H. (2003). Role of nuclear bile acid receptor, FXR, in adaptive ABC transporter regulation by cholic and ursodeoxycholic acid in mouse liver, kidney and intestine. J. Hepatol..

[149] Harrison S.A., Bashir M.R., Lee K.J., Shim-Lopez J., Lee J., Wagner B., Smith N.D., Chen H.C., Lawitz E.J. (2021). A structurally optimized FXR agonist, MET409, reduced liver fat content over 12 weeks in patients with non-alcoholic steatohepatitis. J. Hepatol..

[150] Badman M.K., Chen J., Desai S., Vaidya S., Neelakantham S., Zhang J., Gan L., Danis K., Laffitte B., Klickstein L.B. (2020). Safety, Tolerability, Pharmacokinetics, and Pharmacodynamics of the Novel Non-Bile Acid FXR Agonist Tropifexor (LJN452) in Healthy Volunteers. Clin. Pharmacol. Drug Dev..

[151] Al-Khaifi A., Rudling M., Angelin B. (2018). An FXR Agonist Reduces Bile Acid Synthesis Independently of Increases in FGF19 in Healthy Volunteers. Gastroenterology.

[152] Trauner M., Gulamhusein A., Hameed B., Caldwell S., Shiffman M.L., Landis C., Eksteen B., Agarwal K., Muir A., Rushbrook S. (2019). The Nonsteroidal Farnesoid X Receptor Agonist Cilofexor (GS-9674) Improves Markers of Cholestasis and Liver Injury in Patients with Primary Sclerosing Cholangitis. Hepatology.

[153] Chapman R.W., Lynch K.D. (2020). Obeticholic acid-a new therapy in PBC and NASH. Br. Med. Bull..

[154] Sumida Y., Yoneda M. (2018). Current and future pharmacological therapies for NAFLD/NASH. J. Gastroenterol..

[155] Markham A., Keam S.J. (2016). Obeticholic Acid: First Global Approval. Drugs.

[156] Sun L., Cai J., Gonzalez F.J. (2021). The role of farnesoid X receptor in metabolic diseases, and gastrointestinal and liver cancer. Nat. Rev. Gastroenterol. Hepatol..

[157] Neuschwander-Tetri B.A., Loomba R., Sanyal A.J., Lavine J.E., van Natta M.L., Abdelmalek M.F., Chalasani N., Dasarathy S., Diehl A.M., Hameed B. (2015). Farnesoid X nuclear receptor ligand obeticholic acid for non-cirrhotic, non-alcoholic steatohepatitis (FLINT): A multicentre, randomised, placebo-controlled trial. Lancet.

[158] Food and Drug Administration Ocaliva (Obeticholic Acid) by Intercept Pharmaceuticals: Drug Safety Communication—Due to Risk of Serious Liver Injury, FDA Restricts Use of Ocaliva in Primary Biliary Cholangitis Patients with Advanced Cirrhosis. Posted on 26 May 2021. https://www.fda.gov/safety/medical-product-safety-information/ocaliva-obeticholic-acid-intercept-pharmaceuticals-drug-safety-communication-due-risk-serious-liver.

[159] Ashby K., Navarro Almario E.E., Tong W., Borlak J., Mehta R., Chen M. (2018). Review article: Therapeutic bile acids and the risks for hepatotoxicity. Aliment. Pharmacol. Ther..

[160] Soret P.A., Lam L., Carrat F., Smets L., Berg T., Carbone M., Invernizzi P., Leroy V., Trivedi P., Cazzagon N. (2021). Combination of fibrates with obeticholic acid is able to normalise biochemical liver tests in patients with difficult-to-treat primary biliary cholangitis. Aliment. Pharmacol. Ther..

[161] Zhang T., Feng S., Li J., Wu Z., Deng Q., Yang W., Li J., Pan G. (2022). Farnesoid X receptor (FXR) agonists induce hepatocellular apoptosis and impair hepatic functions via FXR/SHP pathway. Arch. Toxicol..

[162] Lin C., Yu B., Chen L., Zhang Z., Ye W., Zhong H., Bai W., Yang Y., Nie B. (2022). Obeticholic Acid Induces Hepatoxicity Via FXR in the NAFLD Mice. Front. Pharmacol..

